# Diet Quality, Dieting, Attitudes and Nutrition Knowledge: Their Relationship in Polish Young Adults—A Cross-Sectional Study

**DOI:** 10.3390/ijerph19116533

**Published:** 2022-05-27

**Authors:** Marzena Jezewska-Zychowicz, Marta Plichta

**Affiliations:** Department of Food Market and Consumer Research, Institute of Human Nutrition Sciences, Warsaw University of Life Sciences (SGGW-WULS), Nowoursynowska 159 C, 02-776 Warsaw, Poland; marzena_jezewska_zychowicz@sggw.pl

**Keywords:** nutrition knowledge, attitudes, food, dieting, young adults

## Abstract

The purpose of this study was to examine the relationships between diet quality, dieting, nutrition knowledge and attitudes in a group of Polish young adults. A cross-sectional survey was conducted in 2018 amongst 638 students of food and nutrition-related majors. Based on the frequency of consumption of 24 food groups, the “Pro-Healthy Diet Index” (pHDI) and “Non-Healthy Diet Index” (nHDI) were calculated. To assess the nutrition knowledge, the “GAROTA” test was used. The k-means clustering method was used to identify clusters-attitudes towards food and nutrition. The relationships between pHDI and nHDI indices, dieting, nutrition knowledge (NK), and attitudes towards food and nutrition were verified, using multiple linear regression analysis. The results confirmed some relationships between the variables. Higher nHDI characterized males (*p* < 0.0001) and people with more unfavorable attitudes towards food and nutrition (*p* < 0.0001), and those not using a diet (*p* < 0.0001). Higher nutrition knowledge (*p* < 0.0001) and higher BMI (*p* = 0.0370) were correlated with lower nHDI. Higher pHDI characterized people with more favorable attitudes (*p* < 0.0001) and those using a diet (*p* = 0.0002). Nutrition knowledge showed an adverse association with nHDI (*r* = −0.172, *p* < 0.05) and no association with pHDI. Thus, declarative nutrition knowledge does not seem to be a good indicator of healthy dietary behavior. Nutrition education programs that concentrate only on knowledge of facts, and neglect the development of favorable attitudes towards food and nutrition, may not be efficient enough to develop adequate dietary behavior of students.

## 1. Introduction

During the transition from adolescence to adulthood, young people are at a greater risk of engaging in inadequate food habits [1] which, in later life, may increase the odds of developing severe diseases, including osteoporosis, obesity, diabetes, and cancer [2]. Adolescence is viewed as a period in life during which nutrition education can help establish healthy eating habits [3], which will later be maintained throughout life [4]. However, the mechanism by which nutrition knowledge (NK) affects dietary behaviors is complex and non-univocal. Behaviors are determined not only by individuals’ awareness of food, but also by the perceived importance of food for health [5,6].

During the transition from adolescence to adulthood, the acquisition of nutrition knowledge may still be related to schooling, where one acquires information from qualified sources without personal interpretation. Simultaneously, the social environment, including social media, can be an important source of nutrition information and attitudes towards food and nutrition [7]. The growing exposure to web information may contribute to increasing false beliefs [8]. Nevertheless, an individual is also characterized by subjective knowledge [9]. Some studies have shown that a self-perceived level of competency is a stronger driver of behavior than objective knowledge [10,11]. However, an individual with adequate knowledge of nutrition stands a better chance of differentiating nutrition facts from nutrition fads [12], which can affect behaviors and attitudes towards food and nutrition [5,6].

Many studies have reported that university students do not consume an adequate diet [13,14], which can lead to poor health and weight gain [15]. On the other hand, students’ nutrition knowledge varies from poor to satisfactory [16,17]. Previous studies have shown that insufficient knowledge about healthy nutrition causes many inappropriate eating behaviors [18,19], while high nutrition knowledge was significantly associated with healthy eating behaviors, for example, with the intake of fruits, cereals, dairy products, and pulses [20,21,22]. However, studies to date that have addressed the relationship between nutrition knowledge and behaviors have primarily considered the consumption of selected food groups rather than overall diet indices.

Previous studies, both in Europe and other countries, used a variety of tools to measure nutrition knowledge, i.e., the General Nutrition Knowledge Questionnaire (GNKQ) [23] and the Nutritional Knowledge Test (NKT) [4], which was culturally adapted and validated (for example, [24]). Many recent studies have addressed the development and validation of questionnaires for specific nutrition knowledge areas (especially sports nutrition knowledge), or specific populations, e.g., adolescents and athletes (e.g., [25,26]). In Poland, NK assessment was carried out in several studies [27,28], however, most of the employed tools had not undergone a validation process. Although the GAROTA test was developed and validated on a group of Polish students as a tool that can assess their declarative knowledge [29], there are still no published results from studies using this test.

Students majoring in nutrition and medicine are a group of particular interest [17]. The choice of a field of study may be based on the candidate’s interests, so it can be assumed that even the first year students majoring in food and nutrition have a higher level of nutrition knowledge, including declarative knowledge, but may also experience more nutrition problems, such as obsessions or restrictions [30]. As a population group, they distinguish themselves by a relatively higher prevalence of disordered eating behaviors (e.g., high intensity of orthorexic behaviors) or eating disorders (e.g., anorexia) [30,31], which may start with various dietary restrictions. These characteristics justify conducting research in which nutrition knowledge and attitudes towards food and nutrition, but also dieting, are considered as factors explaining the quality of the diet of students involved in food and nutrition-related majors. We hypothesized that students’ nutrition knowledge would correlate positively with their attitudes towards food and nutrition. At the same time, the quality of students’ diets, measured by the healthy diet index (pHDI), would show a positive correlation with both declarative nutrition knowledge and attitudes towards food and nutrition. The unhealthy diet index (nHDI), on the other hand, would show an inverse relationship with these characteristics. We also assumed that the strength of these relationships may not be equal. The purpose of this study was to examine the relationships between nutrition knowledge, attitudes towards food and nutrition, dieting, and the quality of students’ diet, when entering food and nutrition majors. The results obtained can then be applied in the development of educational programs for students that in the future will be responsible for shaping correct eating habits in the rest of the population.

## 2. Materials and Methods

### 2.1. Study Design and Sample Collection

A cross-sectional survey was conducted between January and March 2018 by either a trained person or an academic teacher. Only first-year students of food and nutrition-related majors participated in the study. The selection of universities and participants was made arbitrarily by the researchers. From the list of Polish universities with food and nutrition-related majors, four universities were selected. Selected universities were characterized by a large number of people studying these majors and represented different parts of Poland, i.e., Northern (University of Warmia and Mazury in Olsztyn), Central (Warsaw University of Life Sciences), and Southern (University of Life Sciences in Lublin, and University of Agriculture in Krakow). The study was conducted during lectures and practical classes. All students invited to the study agreed to participate. A paper and pen personal interview (PAPI) technique was used for data collection. In total, 659 students of food and nutrition-related majors gave voluntary consent to participate in the study. Because of missing data in the questionnaires, 21 students were excluded from the sample. The final sample consisted of 638 young adults aged 18–32 years.

### 2.2. Dietary Data

The frequency of consumption of 24 food groups was assessed with the Beliefs and Eating Habits Questionnaire (KomPAN) [32], that was validated in Polish adults [33]. The participants reported the habitual frequency of consuming each food group within the previous 12 months by providing one of the following answers: 1—less than once a month or never; 2—1–3 times a month; 3—once a week; 4—a few times a week; 5—once a day; and 6—a few times a day. During the data analysis, those values were converted to reflect the daily frequency of intake, ranging from 0 to 2 times/day [34].

Two indices were calculated. The first “Pro-Healthy Diet Index” (pHDI) comprised 10 food groups with a potentially beneficial influence on health, while the “Non-Healthy Diet Index” (nHDI), included 14 food groups with a potentially negative influence on health (Table 1).

To standardize the range of both indices, the sum of the frequency of food consumption (times/day) and its expression in scale from 0 to 100 points was calculated. The range of 0–33 points for the pHDI/nHDI index indicates a low adherence to a healthy/unhealthy diet, 34–66 indicates a moderate adherence to a healthy/unhealthy diet, and 67–100 indicates a high adherence to a healthy/unhealthy diet [32].

Moreover, students declared whether they followed a diet (answers: “no”; “yes, on medical recommendation”; and “yes, on own choice”).

### 2.3. Nutrition Knowledge and Attitudes towards Food and Nutrition

The “GAROTA” test was used to measure nutrition knowledge [29]. The test includes a manual that allows the creation of multiple tools by the researcher. The set of questions, of varied difficulty, consists of 14 subject blocks (including energy, carbohydrates, lipids, proteins, minerals, vitamins, anatomy and physiology of the digestive system, metabolism and regulation of food intake, nutritional value of food and its fortification, changes in nutritional value during food processing and storage, nutritional norms and recommendations, nutrition planning and assessment, diet-related diseases and nutrition of sick people, and other). For the test preparation, each time the questions are randomly drawn, according to specific criteria. In each block, there are:

True/false “easy” questions (i.e., “Sucrose is a carbohydrate found in milk”, “The most intensive absorption of digestion products occurs in the large intestine”);

True/false and single-choice “moderately difficult” questions (i.e., “The highest glycemic index is characterized by…”, “The mineral contained in saliva and gastric juice is…”);

Multiple-choice and matching “difficult” questions (i.e., “Plant sterols include…”, “Fats containing unsaturated fatty acids are easily affected by…”).

For this study, the tool contained 42 questions—three questions from each subject block, and each with a varying level of difficulty. The single-choice questions were selected from the “moderately difficult” questions, and the multiple-choice questions from the “difficult” questions. Respondents received one point for each correct answer to all of types of the questions and 0 points for a wrong answer or lack of an answer. The sum of scores ranged between 0 and 42. The higher the score, the more nutrition knowledge the participant had. A high level of nutrition knowledge is attributed to the participant for 80% or more points, a moderate level—60–79%, a low level—40–59%, and an insufficient level—39% or less.

To assess attitudes towards food and nutrition, we used seven statements concerning the links between food, nutrition, and health (four items), as well as food, nutrition, and other life aspects (three items—appearance, self-esteem, and lifestyle) [35]. Items were scored as follows: 1—always; 2—often; 3—sometimes; and 4—never. The sum of scores ranged between 7 and 28. The higher the score, the less favorable was the attitude displayed towards food and nutrition.

### 2.4. Sociodemographic Data

The sociodemographic profile of the participants was assessed based on questions related to the following variables: gender; age (in years); place of residence (village, town with less than 100,000 inhabitants, city with above 100,000 inhabitants). The body mass index (BMI) of the participants was calculated from the self-reported body weight and height. The participants were categorized into three groups, based on their BMI, according to the classification of World Health Organization [36]: underweight (BMI < 18.5 kg/m^2^); of normal weight (BMI between 18.5 and 24.9 kg/m^2^); overweight and obese (BMI ≥ 25.0 kg/m^2^).

### 2.5. Statistical Analysis

Descriptive statistics were used to present the sociodemographic characteristics of the study sample. Data were presented as a sample percentage (%) for categorical data or mean and standard deviation (SD) for continuous data. The normality of the distribution of continuous variables was assessed with the normality Kolmogorov–Smirnov test, the Lilliefors test, and normal probability plot. 

The k-means clustering method was used to identify clusters (attitudes towards food and nutrition) according to seven statements concerning links between food, nutrition, health, and other life aspects. In the k-means method, the average values for individual clusters obtained using the hierarchical method were used as seeds. Two separate clusters representing different attitudes towards food and nutrition were obtained, which was confirmed by both statistics’ assessment of the selection of clusters, such as cubic clustering criteria–CC = 27.5, pseudo F = 223, and ANOVA (analysis of variance) statistics comparing the average values of variables for individual clusters (*p* < 0.001 for all of the variables). Cluster 1 represented “favorable attitudes” towards food and nutrition, while in cluster 2 “unfavorable attitudes” were observed (Figure S1). The independence χ^2^ test was used to assess the clusters’ profiles, according to the sociodemographic characteristics. The Student’s *t*-test was used to compare mean values of beliefs between clusters. 

The associations between the scores of the pHDI and nHDI indices, dieting, nutrition knowledge, and attitudes towards food and nutrition were verified using multiple linear regression analysis, adjusted for gender (categorical, female/male), age (continuous, in years), place of residence (categorical, village/town < 100,000 inhabitants/city ≥ 100,000 inhabitants), and BMI (continuous, kg/m^2^). The pHDI and nHDI scores, treated as continuous variables, were introduced into the models separately as a dependent variable. The independent variables included in the models were: using a diet (categorical, yes/no); nutrition knowledge (continuous, points); and attitudes towards food and nutrition (continuous, points). Two models were created as follows: model 1—adjusted for the set of confounders for nHDI score; and model 2—adjusted for the same confounders, as in model 1 and pHDI score. Automatic selection of the variables with the forward selection method was applied to the models. Standardized regression coefficient (*β*), and unstandardized regression coefficient (*B*) with a 95% confidence interval (CI) were used. Significance was set at *p* < 0.05 for all of the analyses. Analysis conducted on the Model 1 and Model 2 explained, respectively, 16% and 14% of variance (R^2^ = 0.16; R^2^ = 0.14, respectively). The Durbin–Watson test was used to check the assumption of no correlation of the random components. The tolerance statistic was applied to assess the assumption of no correlation of the predictors. The Durbin–Watson statistics were 1.98 for Model 1, and 2.01 for Model 2, which means that the assumption was fulfilled. The tolerance statistics were above 0.1 for both models, thus the assumption about a lack of correlation between the predictors was also fulfilled. Significance was confirmed at *p* < 0.05. The analyses were carried out using IBM SPSS Statistics for Windows, version 26.0 (IBM Corp, Armonk, NY, USA).

## 3. Results

### 3.1. Characteristics of the Study Sample

Table 2 presents the sociodemographic characteristics of the participants. The sample consisted of 638 students (514 women and 124 men), aged 18–32 years. Normal body weight characterized 76.1% of the study group.

### 3.2. Nutrition Knowledge and Diet Indices

The mean score of nutrition knowledge was 18.6 points, or 44.3% of the maximum score (42 points), which, according to the accepted criteria in the GAROTA test, indicates a low level of nutrition knowledge. The highest score attained (31 points) corresponded to 73.8% of the maximum score, which means a moderate level of nutrition knowledge [29].

In the study group, 153 people (24%) were currently using a diet. In the group declaring the use of a diet, 24.4% were on an elimination diet, 19.3% on a low-calorie diet, 17.6% on a high-protein diet, and 16% on a vegetarian diet. The mean values of both indices describing dietary characteristics, i.e., pHDI and nHDI, were below 33 points. Thus, the study subjects were characterized by a low intensity of both healthy and unhealthy diets (Table 3). 

The associations between nutrition knowledge, attitudes, and diet indices are shown in Table 4. Nutrition knowledge did not correlate with the pro-healthy diet index (pHDI). In contrast, it correlated negatively with the unhealthy diet index (nHDI), i.e., greater nutrition knowledge was associated with a lower intensity of unhealthy diet. Attitudes towards food and nutrition correlated positively with the pHDI, i.e., a more favorable attitude was associated with a higher intensity of healthy diet, and adversely with the nHDI; BMI and age did not correlate with nutrition knowledge, attitudes, and diet indices. 

### 3.3. Attitudes towards Food and Nutrition—Identified Clusters

Most people surveyed confirmed the statements that eating healthy foods can improve their appearance and that food choices are determined by concern for their health. In contrast, the respondents were least likely to describe themselves as people who pay attention to the caloric value of the foods they eat (Table 5).

Based on the participants’ opinions about the links between food, nutrition, and health (four items), as well as between food, nutrition, and other aspects of life (three items) two clusters were identified (Table 5). Statistically significant (*p* < 0.05) differences between both clusters were observed in all of the analyzed statements. In general, lower mean values for all of the statements (range between “always” and “often”) were observed in cluster 1 compared to cluster 2 (range between “often” and “sometimes”). Therefore, individuals in cluster 1 represented more favorable attitudes towards food and nutrition, compared to those in cluster 2 (*p* < 0.001).

### 3.4. Profiles of Identified Clusters

Sociodemographic variables such as gender, age, education, and place of residence were used to profile the clusters. However, no significant differences were found between clusters for these variables, except for a place of residence (Table 6). More people living in the rural environment adhered to cluster 2 and more people living in an urban environment adhered to cluster 1. There were no differences between both of the clusters, after taking BMI into account.

People from cluster 1 had higher nutrition knowledge than the others (Table 6). They had a higher pHDI and lower nHDI, while those from cluster 2 had a lower pHDI and higher nHDI.

### 3.5. Relationships between Nutrition Knowledge, Attitudes towards Food and Nutrition, Dieting, and Diet Indices

The results of the multiple linear regression analysis that examined the relationship between nHDI, dieting, nutrition knowledge, and attitudes towards food and nutrition are shown in Table 7. The nHDI showed associations with nutrition knowledge, attitudes towards food and nutrition, using a diet, gender, and BMI. The higher nHDI characterized males and people with a higher score for attitudes towards food and nutrition (a more unfavorable attitude) and those not using a diet. By contrast, higher nutrition knowledge and higher BMI were both correlated with lower nHDI.

The pHDI showed inverse associations with attitudes towards food and nutrition, as well as not using a diet (Table 7). A higher pHDI characterized people with a lower score for attitudes towards food and nutrition (more favorable attitude), and those using a diet.

## 4. Discussion

### 4.1. Nutrition Knowledge

The GAROTA test indicated a low level of nutrition knowledge of students entering food and nutrition-related majors, which is not surprising. Such results are, on the one hand, due to the specificity of the test, in which the questions relate to the level of knowledge offered in the programs of technical secondary schools and universities, as well as available in school and academic textbooks on human nutrition [29]. On the other hand, the nutrition knowledge of young people is not always satisfactory. Previous studies conducted on students from different countries (i.e., Poland, Australia, Great Britain, Kuwait, Ghana) showed inconsistent results. It was demonstrated that nutrition knowledge was satisfactory for 34.7% of women and 40.2% of men, while it was good for 34.7% of women and 25.1% of men, respectively [37]. Other studies determined the level of their knowledge as good [38,39], below average [40], but also, as in our study, the nutrition knowledge was assessed as low [41,42]. The inconsistent results obtained in different studies may result from the field of the study, but also from the specificity of the measurement tools [37,42]. A higher similarity of the results was obtained from the studies that used the same tool, for example, the General Nutrition Knowledge Questionnaire (GNKQ) [43,44]. However, the use of the GNKQ gave distinct results in different countries, e.g., Kuwait [42] and Great Britain [39], which may confirm the importance of socio-cultural differences in determining the different results obtained. Measuring nutrition knowledge in groups with different cultural backgrounds, especially with the use of different tools, makes it difficult to compare the results between groups, but it does not negatively influence the possibility of studying the relationship between nutrition knowledge, attitudes, and behaviors. Nevertheless, further cultural adaptations and validation of tools employed to measure nutrition knowledge would provide an opportunity to learn about the level of knowledge, and subsequently its relationship to a diet, regardless of socio-cultural context [45].

### 4.2. Diet Indices

The participants were characterized by a low intake of food with both potentially beneficial (pHDI) and adverse influences on health (nHDI). Other studies showed that the diet of young people is characterized by many nutritional irregularities [37,46], and therefore low nHDI can be considered as a positive outcome. Nevertheless, the low nHDI accompanied by the low pHDI indicates the existence of a problem with inadequate food intake in college students, as confirmed by other researchers [13,14]. In our study, the pHDI index correlated positively with age, whereas the nHDI index was higher among males compared to females. In the previous study, the diet quality index was also worse among younger people and men [47]. The reason may be that women show a more specific attitude towards food and nutrition, due to their desire to achieve or maintain a slim body [48]. Moreover, people using different types of vegetarian diets (i.e., lacto-ovo vegetarian, vegan, pesco-vegetarian, semi-vegetarian) had higher diet quality indices compared to people not using these diets [49]. Similarly, in our study, the pHDI index was associated with the use of the diet, also including vegetarian diets, while the nHDI was associated with not using such diets. This can be associated with a concern about the quality of the food consumed and choosing the right food, which in turn affects the better quality of the diet [50].

### 4.3. Diet Indices and Nutrition Knowledge

In our study, nutrition knowledge correlated negatively with the unhealthy diet index (nHDI), i.e., greater nutrition knowledge was associated with a lower intensity of unhealthy diets (model 1). This is supported by the findings of other studies, indicating that students with greater nutrition knowledge consumed less unhealthy fats and cholesterol [19], and ate more vegetables and fruit as a snack instead of salty snacks [37]. Thus, the expectations that nutrition knowledge and understanding of the importance of dietary habits of the students of food and nutrition-related majors would correlate with more desirable behaviors were confirmed [51]. At the same time, low knowledge was shown to correlate with poor dietary behavior [52]. In contrast, nutrition knowledge did not correlate with the pro-healthy diet index (pHDI) (model 2). In other studies, nutrition knowledge in university students was found to associate positively with an increased intake of fruit, dairy, protein, and wholegrain foods [53], as well as with a higher likelihood to meet dietary recommendations [54], or other positive dietary behaviors (i.e., reading food labels) [43]. The lack of correlation between nutrition knowledge and a healthy diet in our study may suggest that declarative knowledge may not convert into skills or procedural knowledge, that is, the ability to choose healthier food, understand food labels, or choose healthier options from available food [22]. It seems that the term “nutrition literacy”, defined as the degree to which individuals can obtain, process, and understand nutrition information and the skills needed to make good nutrition decisions [55], will help individuals gain a better understanding of the link between “knowing” and “behaving”. Thus, future studies should include a tool that also assesses procedural knowledge among food and nutrition students, to better understand the relationship between their nutrition knowledge, attitudes towards food and nutrition, and diet. The lack of a relationship between declarative nutrition knowledge and pHDI, while there was a confirmed relationship between attitudes and pHDI, suggests the need for further research to confirm whether this could be determined by only measuring declarative knowledge. However, further research should consider not only procedural nutrition knowledge, but also the affective component of attitudes toward food and nutrition.

One of the other explanations for the results obtained may be the fact that the study group consisted of first-year students. Their characteristics may therefore be closer to those of the general secondary school students’ population [56], and not the entire student population. A study by Spexoto et al. (2015) [57] found that among the students of the first year of university low attention to diet correlated with poorer diet quality, as well as with low physical activity. Enrolling in nutrition-related studies increases nutrition knowledge significantly, as evidenced by the differences in nutrition knowledge between students of these majors and others [58]. Nutrition education may change both students’ dietary habits [59], and healthy lifestyle choices [60]. There could be, however, many other factors that impinge on students’ dietary behaviors [61,62]. Thus, longitudinal studies that include these factors are recommended to understand the relationship between increasing nutrition knowledge and changes in eating behavior.

### 4.4. Attitudes towards Food and Nutrition, Nutrition Knowledge, and Diet Indices

The respondents thought that eating healthy foods could improve their appearance, but they also linked their food choices with a concern about their health, which is confirmed by other studies conducted in the group of young people [63,64]. The study group showed little variation in the opinions regarding the association of food with health and other aspects of life (self-esteem, lifestyle, physical attractiveness), which may result from the high homogeneity of the study sample caused by age and student status. However, the two groups that were distinguished according to attitudes to food and nutrition showed significant differences from each other. It was found that people from cluster 1 (favorable attitude) were more engaged in the relationship of food with both health and other aspects of life. This holistic approach to food in this group may translate to treating food as an important life value, and at the same time linking it to many dimensions of life, i.e., health, self-esteem, and lifestyle. This may explain the lack of correlation between nutrition knowledge and pHDI and confirm that eating behaviors are determined by many factors, and nutrition knowledge is only one of them [61,62].

Consistent with previous research [65], nutrition knowledge differentiated attitudes toward food and nutrition (clusters). Those for whom the relationship of food with both health and other aspects of life was more important (cluster 1) had greater nutrition knowledge. Thus, they were more aware of the health effects of the food they consumed, but also attributed greater importance to food and nutrition for their self-esteem, lifestyle, and physical attractiveness. These individuals had a lower intensity of unhealthy diet and a higher intensity of healthy diet. The inclination to healthy eating is perceived as a desirable behavior in general. Nevertheless, the concern should arise when food determines the individual’s self-esteem and physical attractiveness, as healthy eating can take on obsessive qualities and prevent a person from leading a healthy life, and even lead to disordered eating behaviors, such as an obsession with healthy eating [66].

Based on the obtained results, we verified the hypotheses assuming that nutrition knowledge correlates positively with attitudes toward food and nutrition, and that an unhealthy diet shows a relationship with lower nutrition knowledge and unfavorable attitudes towards food and nutrition. The healthy diet index correlated positively with favorable attitudes, whereas the positive correlation between nutrition knowledge and pHDI was not verified either by two-way correlation, or in a linear regression model. The strength of the relationships between the variables was weak. The results confirmed the stronger relationship of favorable attitudes towards food and health with diet quality (lower nHDI and higher pHDI) than nutrition knowledge alone (only correlation with nHDI). These findings could be used in strategies being developed for young people in Poland, but also in other countries, which justifies the high similarity of specificity of this age group, e.g., widespread use of mass communication, social media, dietary mistakes, eating disorders, etc.

### 4.5. Limitations

The limitation of the study was that the study group was not representative of the Polish student population as a whole, but only of the students of food and nutrition-related majors, representing four of the largest universities which offered such courses. Thus, generalizations and extrapolations at a broad level are not permitted. BMI was calculated based on self-reported data. Moreover, it concerned a specific group, i.e., students of nutrition, and did not reflect the available data of the population of young Poles. In 2019, the 20–29 age group of Poles had fewer underweight people (6.3% vs. 10.3 in the study sample), fewer normal weight people (58.7% vs. 76.1%), and more overweight and obese people (35.2% vs. 13.6%) [67], that can be explained by the specificity of the study group, which included only students of the majors related to food and nutrition, and therefore people who are interested in nutrition and body weight, which may explain their lower body weight compared to the population of young Poles aged 20–29 [67]. Another limitation was related to the results of the multiple linear regression models, which accounted for only 16% (Model 1), and 14% (Model 2) of the variance of the dependent variable. Lastly, this was a cross-sectional study and did not allow us to assess the causality of relationships between the variable.

## 5. Conclusions

The study aimed to examine the relationships between nutrition knowledge, attitudes towards food and nutrition, dieting, and the quality of diet of students entering food and nutrition-related majors; these are particularly important because of their role in shaping proper eating habits among people in the future. The results obtained confirmed the relationship between nutrition knowledge and attitudes towards food and nutrition, whereas the association of these constructs with eating behaviors showed a different character. Nutrition knowledge showed an adverse association with nHDI and no association with pHDI, whereas more positive attitudes towards food and nutrition were associated with a higher pHDI and a lower nHDI, i.e., a more adequate diet. Thus, the findings suggested that declarative nutrition knowledge does not seem to be a good indicator of healthy dietary behavior, especially when nutrition knowledge is estimated to be low. Students completing nutrition majors will gain factual nutrition knowledge, however, there is a need to develop their positive attitudes and the skills that can bring about behavioral change. Nutrition education programs that concentrate only on knowledge of the facts, or so-called declarative knowledge, and that neglect the development of favorable attitudes towards food and nutrition, may not be efficient enough to develop adequate eating behaviors of the students, who will provide nutrition counseling in the future and thus shape the correct eating habits in the rest of population.

## Figures and Tables

**Table 1:** Components of the diet indices.

**“Pro-Healthy Diet Index” (pHDI)—Food Groups:**	
1.	wholemeal bread, wholemeal bread rolls
2.	buckwheat, oats, wholegrain pasta, or other coarse-ground groats
3.	milk
4.	fermented milk beverages (e.g., yoghurts, kefir)
5.	fresh cheese curd products (e.g., cottage cheese, homogenized cheese, fromage frais)
6.	white meat (e.g., chicken, turkey, rabbit)
7.	fish
8.	pulse-based foods (e.g., from beans, peas, soybeans, lentils)
9.	fruit
10.	vegetables
**“Non-Healthy Diet Index” (nHDI)—Food Groups:**	
1.	white bread and bakery products (e.g., wheat bread, toast bread, white bread rolls)
2.	white rice, white pasta, fine-ground groats (e.g., semolina, couscous)
3.	fast foods
4.	fried foods (e.g., meat or flour-based foods such as dumplings, pancakes, etc.)
5.	butter (as a bread spread or as an addition to meals for frying, baking, etc.)
6.	lard
7.	cheese (including processed cheese, blue cheese)
8.	cold meats, smoked sausages, hot-dogs
9.	red meat (e.g., pork, beef, veal, mutton, lamb, game)
10.	sweets
11.	tinned meats
12.	sweetened carbonated or still beverages
13.	energy drinks
14.	alcoholic beverages

**Table 2:** Characteristics of the study sample.

	Total	
	*N* *	%
Total	638	100.0
Gender		
Women	514	80.4
Men	124	19.6
Age		
18–24 years old	383	60.1
25–32 years old	255	39.9
Place of residence		
Village	278	43.5
A town with less than 100,000 inhabitants	160	25.2
A city with over 100,000 inhabitants	200	31.3
BMI (kg/m^2^)		
Underweight (BMI < 18.5)	66	10.3
Normal weight (BMI 18.5–24.9)	485	76.1
Overweight and obese (BMI ≥ 25)	87	13.6
Age (mean; SD in years)	20.7; 1.4	
BMI (mean; SD in kg/m^2^)	21.8; 3.3	

* N—number of participants.

**Table 3:** Participants’ nutrition knowledge, attitudes, and diet indices.

Variables	Mean; SD	Min.–Max.
Nutrition knowledge	18.6; 3.63	8–31
Attitudes towards food and nutrition	15.6; 3.86	7–28
Pro-Healthy Diet Index (pHDI)	25.9; 11.63	3.4–85.7
Non-Healthy Diet Index (nHDI)	16.3; 9.30	0–75.2

**Table 4:** Correlations between nutrition knowledge, diet indices, BMI, and participants’ age.

	Nutrition Knowledge	Attitudes towards Food and Nutrition	pHDI	nHDI	BMI	Age
Nutrition knowledge (in points)	1	−0.079 *	0.039	−0.172 **	−0.020	0.063
Attitudes towards food and nutrition (in points)		1	−0.342 **	0.299 **	0.012	−0.063
Pro-Healthy Diet Index (pHDI)			1	0.006	0.060	0.098
Non-Healthy Diet Index (nHDI)				1	−0.011	−0.059
BMI (kg/m^2^)					1	0.169 **
Age (in years)						1

* Significant at *p* < 0.05; ** at *p* < 0.01 (Pearson’s correlation).

**Table 5:** Characteristics of the identified clusters according to the classification variables.

Items	Total	Attitudes towards Food and Nutrition		
		“Favorable Attitudes” CLUSTER 1	“Unfavorable Attitudes” CLUSTER 2	Sig.
	Mean *; SD	Mean; SD	Mean; SD	
I pay attention to the caloric value of the foods I eat	2.5; 0.8	2.1;0.8	2.9; 0.7	<0.001
Thinking about food is a particular concern for me	2.4; 0.9	2.1; 0.8	2.8; 0.8	<0.001
My food choices are determined by concern for my health	2.2; 0.8	1.8; 0.7	2.6; 0.7	<0.001
I am willing to spend more money to buy healthy food	2.3; 0.8	2.0; 0.7	2.7; 0.7	<0.001
A belief in healthy eating increases my self-esteem	2.4; 1.0	1.8; 0.8	3.0; 0.8	<0.001
Healthy eating influences my lifestyle	2.3; 1.0	1.7; 0.7	3.0; 0.8	<0.001
Eating healthy foods can improve my appearance	1.5; 0.7	1.2; 0.5	1.9; 0.8	<0.001
Attitude towards food and nutrition—sum of scores	15.6; 3.9	12.7; 2.3	18.9; 2.5	<0.001

* A four-point scale: 1—always; 2—often; 3—sometimes; 4—never; SD—standard deviation, Sig.—the Student’s *t*-test.

**Table 6:** Characteristics of the clusters.

	Attitudes towards Food and Nutrition			
	“Favorable Attitudes” CLUSTER 1		“Unfavorable Attitudes” CLUSTER 2	
	*N*	%	*N* *	%
Total	342	53.6	296	46.4
Gender (ns) *				
Women	273	53.1	241	46.9
Men	69	55.6	55	44.4
Age (ns) *				
18–24 years old	205	53.5	178	46.5
25–32 years old	137	53.7	118	46.3
Place of residence (*p* = 0.035) *				
Village	134	48.2	144	51.8
A town with less than 100,000 inhabitants	88	55.0	72	45.0
A city with over 100,000 inhabitants	120	60.0	80	40.0
Using the diet (*p* < 0.001) *				
No	226	46.6	259	53.4
Yes	116	75.8	37	24.2
BMI (kg/m^2^) (ns) *				
Underweight (BMI < 18.5)	32	48.5	34	51.5
Normal weight (BMI 18.5–24.9)	261	53.8	224	46.2
Overweight and obese (BMI ≥ 25)	49	56.3	38	43.7
Age (mean; SD in years) (ns) **	20.7; 1.7		20.6; 1.4	
BMI (mean; SD in kg/m^2^) (ns) **	21.9; 3.3		21.8; 3.4	
Nutrition knowledge (mean; SD in points) (*p* = 0.02) **	18.9; 3.9		18.2; 3.3	
pHDI (mean; SD in points) (*p* < 0.001) **	28.8; 11.6		22.7; 10.7	
nHDI (mean; SD in points) (*p* < 0.001) **	14.5; 8.2		18.3; 10.0	

*N*—number of participants; *p*—significance; ns—no statistical differences between clusters; * χ^2^ test ** Student’s *t-*test; SD—standard deviation; pHDI—Pro-Healthy Diet Index; nHDI—Non-Healthy Diet Index.

**Table 7:** Multiple linear regression analysis of nHDI and pHDI in the study sample.

Parameter	*B*	*β*	95% CI	*p*-Value
	Adjusted Model 1—nHDI			
BMI	−0.23	−0.08	−0.16; −0.00	0.0370
Nutrition knowledge	−0.36	−0.14	−0.21; −0.07	0.0001
Attitudes towards food and nutrition	0.59	0.25	0.17; 0.32	<0.0001
Male (ref. female)	2.58	0.22	0.14; 0.29	<0.0001
Not using the diet (ref. using the diet)	1.32	0.12	0.04; 0.19	<0.0001
	Adjusted Model 2—pHDI			
Not using the diet (ref. using the diet)	−1.97	−0.14	−0.22; −0.07	0.0002
Attitudes towards food and nutrition	−0.88	−0.29	−0.36; −0.21	<0.0001

*B*—unstandardized regression coefficient; *β*—standardized regression coefficient; (95% CI)—95% confidence interval (ref.)—reference group; Adjusted for gender (categorical, female/male); age (continuous, in years); place of residence (categorical, village/town < 100,000 inhabitants/city ≥ 100,000 inhabitants); BMI (continuous, kg/m^2^).

## Data Availability

Due to ethical restrictions and participant confidentiality, data cannot be made publicly available. However, data from the study are available upon request, for researchers who meet the criteria for access to confidential data. Data requests can be sent to the project leader (Marta Plichta).

## Supplementary Materials

The following supporting information can be downloaded at: https://www.mdpi.com/article/10.3390/ijerph19116533/s1, Figure S1: The cluster center plot for attitudes towards food and nutrition.
