# Supplementary figures and images for: Diet Quality, Dieting, Attitudes and Nutrition Knowledge: Their Relationship in Polish Young Adults—A Cross-Sectional Study
Source: Int J Environ Res Public Health. 2022 May 27;19(11):6533. doi: 10.3390/ijerph19116533 (PMC9180766; doi:10.3390/ijerph19116533)

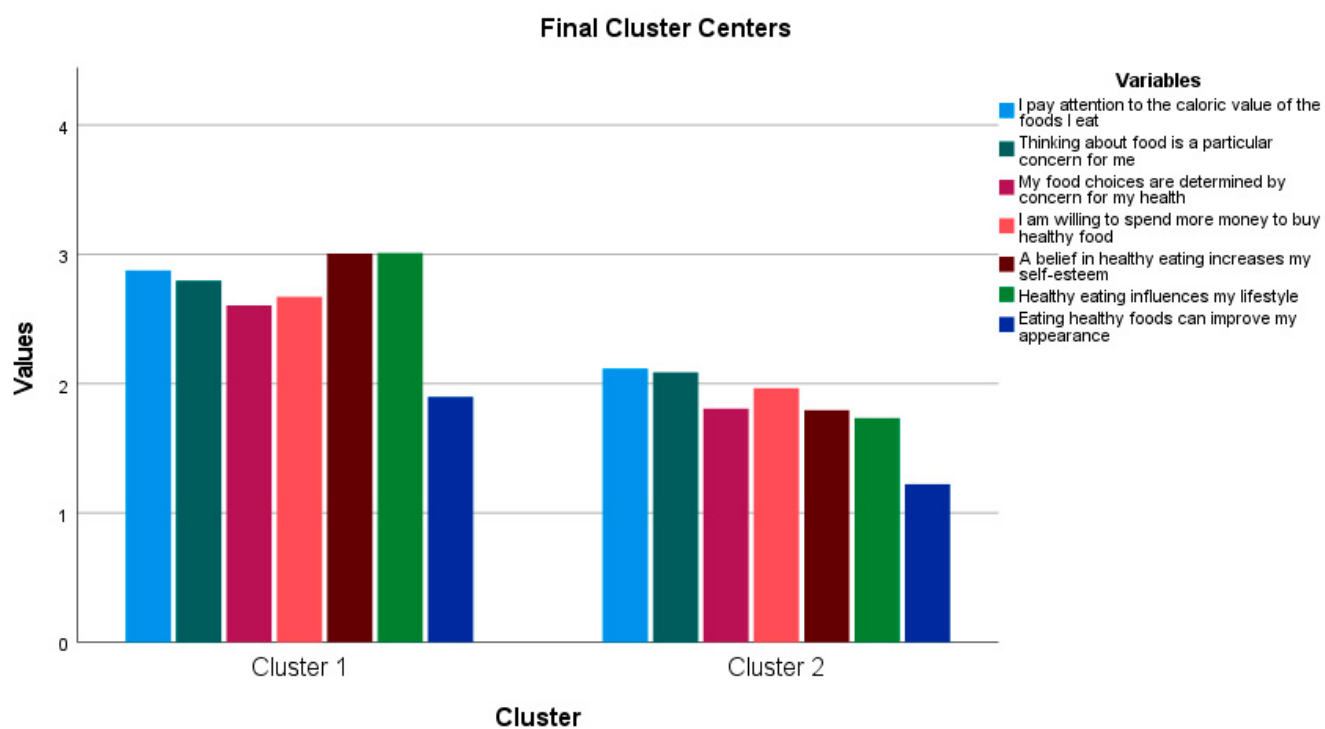

**Figure S1.** The cluster center plot for attitudes towards food and nutrition.

Supplement: Supplementary file 1 [file ijerph-19-06533-s001.zip › ijerph-1710363-supplementary.pdf]
